# A qualitative interview study investigating patient, health professional, and developer perspectives on real-world implementation of patient-centered AI systems

**DOI:** 10.1038/s41746-026-02587-5

**Published:** 2026-05-05

**Authors:** Natalie Benda, Pooja Desai, Zayan Reza, Victoria Winogora, Uday Suresh, Yiye Zhang, Alison Hermann, Rochelle Joly, Jyotishman Pathak, Meghan Reading Turchioe

**Affiliations:** 1 School of Nursing, Columbia University, New York, NY USA; 2 Department of Biomedical Informatics, Columbia University, New York, NY USA; 3 Mailman School of Public Health, Columbia University, New York, NY USA; 4 Department of Biomedical Informatics, Vanderbilt University Medical Center, Nashville, TN USA; 5 Department of Population Health Sciences, Weill Cornell Medicine, New York, NY USA; 6 Department of Psychiatry, Weill Cornell Medicine, New York, NY USA; 7 Department of Obstetrics and Gynecology, Weill Cornell Medicine, New York, NY USA

**Keywords:** Health care, Mathematics and computing, Medical research, Scientific community

## Abstract

Artificial intelligence (AI) systems in healthcare often fail to improve patient outcomes despite high development accuracy. We conducted semi-structured interviews with patients (*n* = 18), health professionals (*n* = 8), and AI developers (*n* = 8), using a postpartum depression risk algorithm as a use case. Through thematic analysis informed by sociotechnical frameworks, we identified six themes: harm mitigation, clinical utility, communication strategies, data quality, privacy/security, and responsible governance. All stakeholders emphasized that patient-centered AI must provide actionable benefits while minimizing bias, stigma, and anxiety. Patients wanted professional interpretation of AI outputs. Participants identified tensions between explainability and accuracy, varying patient preferences for accessing predictions, and unclear accountability when AI recommendations cause adverse outcomes. Our findings support patient-centered implementation through four strategies: providing professionals with competencies and protected time; engaging stakeholders throughout development; offering flexible communication accommodating diverse health literacy; and establishing multi-layered governance with shared accountability across developers, professionals, and institutions.

## Introduction

The transition to artificial intelligence (AI) in healthcare continues to be significant for patients and health professionals^1–4^. Despite AI’s predictive and generative power, many systems are never implemented or, when implemented, struggle to show improvements in patient outcomes compared to the standard of care^5^. This clinical translation gap between AI development and real-world, successful deployment is defined as the “last mile” problem. The “last mile” problem is the fact that AI models may show high accuracy and promise in simulated lab-based tasks, but (1) they are either never deployed in clinical environments, or (2) they cannot improve outcomes in real-life due to deficiencies in implementation. A critical gap lies in end users seeing, accepting, and acting upon AI-based recommendations^6^. Previous research in human-AI interaction has focused heavily on health professionals as the sole recipients of AI recommendations, with less attention to implementing patient-centered AI systems^7^. Our previous pre-implementation work for AI-based systems highlighted the importance of keeping patients informed regarding AI-informed recommendations to justify interventions and to support uptake^8^.

Patients and the public have important perspectives regarding AI implementation and want to be part of the conversation^9–11^. However, their perspectives on centering patients in AI system implementation have been understudied compared to health professionals and AI developers^7,8,12–17^. Moreover, previous studies have independently surveyed patient, health professional, and developer preferences about AI implementation. No studies to our knowledge, however, have collected richer qualitative data and combined their collective insights. Consequently, we lack integrative, practical guidance for those seeking to ethically implement and evaluate patient-centered AI systems.

It is also becoming more likely that patients may access AI output, even if not intended by AI developers. The U.S. 21st Century Cures Act, for example, prevents information blocking from patients, requiring organizations to give patients access to their electronic health information without delay or expense^18^. This may result in a patient seeing an AI-based risk prediction before discussing it with their healthcare team. AI is also increasingly used in tools to support shared decision-making with patients^19^. While AI output may benefit patients, it may cause stress if not appropriately contextualized. AI can also make mistakes, which may be challenging for patients and health professionals to detect.

Recent systematic reviews have examined stakeholder perspectives on AI in healthcare, but significant gaps remain. A scoping review of communicating predictive model results between providers and patients found only 10 studies meeting the inclusion criteria. Moreover, most did not focus on patients’ unique needs^7^. Another systematic review of AI-enabled decision aids for shared decision-making similarly revealed limited evidence, with most articles focused on screening and prevention rather than treatment contexts^19^. A qualitative meta-synthesis of public perceptions identified important concerns about AI reliability, data ethics, and responsibilities, but noted that studies examining multiple stakeholder perspectives simultaneously remain rare^11^. Importantly, no prior work has integrated qualitative insights from patients, health professionals, and developers within a unified framework to understand how AI-based predictions should be communicated and acted upon in clinical practice.

Our objective was to triangulate perspectives from patients, developers, and health professionals for real-world implementation of patient-centered AI systems. We interviewed these three stakeholder groups using interview guides informed by bioethics and informatics models^20^. By integrating perspectives from all three groups within a single socio-technical framework, our study surfaces implementation tensions, such as divergent views on accountability, accuracy-explainability tradeoffs, and communication preferences, that are not visible in single-stakeholder designs. To concretize discussions, we used an existing predictive AI tool developed by our team that proactively predicts a pregnant patient’s risk of postpartum depression (PPD) as a use case. PPD presents a complex bioethical case because of the sensitivity of the data and the need for multi-layer autonomy. In pregnancy, the autonomy, harms, and benefits afforded to the perinatal patient, newborn, or fetus, and partner must be weighed simultaneously. Recruited patients and health professionals were potential users of the tool. We deliberately chose a complex use case that interacts with various domains and considerations. PPD presents a complex bioethical case because of the sensitivity of the data and for the multi-layer autonomy; in pregnancy, the autonomy, harms, and benefits to the perinatal patient, newborn, or fetus, and partner must be weighed simultaneously, invoking all aspects of a biopsychosocial conceptualization of patient care^11,12,21^. Our study provides novel, data-driven recommendations for how AI may be ethically implemented to support patient-centered care through a multi-faceted, contextually rich use case.

## Results

Table 1 provides demographic data from each stakeholder group (*N* = 36). We first deductively coded data based on Sittig and Singh’s Socio-technical Model for Studying Health Information Technology in Complex Adaptive Healthcare Systems, then inductively derived emerging themes for implementing patient-centered AI systems that cut across our overarching framework for analysis (Table 2)^20^. We describe the detailed emerging themes with the most illustrative quotes from developers (DV), health professionals (HP), and patients (PT), including a participant number to demonstrate the breadth of perspectives.

**Table 1:** Participants’ demographics provided at the aggregate level and broken out by stakeholder group

Demographic characteristic		Stakeholder group			
		Overall (*N* = 36), *n* (%)	Developers (*n* = 10), *n* (%)	Health Professionals (*n* = 8), *n* (%)	Patients (*n* = 18), *n* (%)
Sex	Female	29 (81)	5 (50)	7 (87.5)	17 (94.5)
	Male	4 (11)	4 (40)	0 (0)	0 (0)
	Missing	3 (8)	1 (10)	1 (12.5)	1 (5.5)
Race	White	13 (36)	5 (50)	2 (25)	6 (33.5)
	Asian	6 (17)	4 (40)	2 (25)	0 (0)
	Black or African American	9 (25)	0 (0)	2 (25)	7 (39)
	Native Hawaiian or Other Pacific Islander	1 (3)	0 (0)	0 (0)	1 (5.5)
	More than One Race	2 (5.5)	0 (0)	0 (0)	2 (11)
	Prefer Not to Answer	2 (5.5)	0 (0)	1 (12.5)	1 (5.5)
	Missing	3 (8)	1 (10)	1 (12.5)	1 (5.5)
Age (generation)	Generation Z (1997–2012)	6 (17)	3 (30)	0 (0)	3 (17)
	Millennials (1981–1996)	23 (64)	5 (50)	5 (62.5)	13 (72)
	Generation X (1965–1980)	3 (8)	0 (0)	2 (25)	1 (5.5)
	Boomers (1946–1964)	1 (3)	1 (10)	0 (0)	0 (0)
	Missing	3 (8)	1 (10)	1 (12.5)	1 (5.5)
Education	Some College or Associate’s Degree	6 (17)	0 (0)	0 (0)	6 (33.5)
	Bachelor’s Degree	11 (31)	1 (10)	2 (25)	8 (44)
	Master’s Degree	8 (22)	2 (20)	3 (37.5)	3 (17)
.	Doctorate Degree	8 (22)	6 (60)	2 (25)	0 (0)
	Missing	3 (8)	1 (10)	1 (12.5)	1 (5.5)

**Table 2:** Emerging themes from our analysis and their intersection with the dimensions of Sittig and Singh’s Socio-technical framework^a^

	Sittig and Singh dimension							
Emerging theme	Software, hardware, and computing infrastructure	Clinical content	Human- computer interface	Workflow and communication	People	Internal organizational policies, procedures, and culture	External rules, regulations, and pressures	System measurement and monitoring
Mitigating the harms of AI to end users is critically important	✓	✓		✓	✓		✓	✓
AI tools must provide clinical benefit and utility to patients, health professionals, or other end users by making recommended actions clear and easily implementable	✓		✓		✓	✓		✓
Patients should be considered in the communication of AI models, but additional work will be needed to do this effectively	✓	✓	✓	✓	✓			
Data security, privacy, and access should prioritize safeguarding patients	✓				✓	✓	✓	
Health professionals, health systems, and developers were all perceived as having a role in enacting responsible AI practices and assuming liability					✓	✓	✓	✓
Health professionals raised concerns about data quality issues that may lead to less accurate predictions for patients.	✓	✓		✓				

^a^Checkmarks indicate that the theme was substantively addressed in relation to the corresponding dimension.

### Mitigating harm

All participant roles stressed the importance of ensuring minimal harm to end users (e.g., patients, health professionals) related to AI. Perceived harms related to stigma in predicting sensitive health issues, model bias, and potential stress predictions caused to patients.

All stakeholder groups discussed stigmas driven by AI-based predictions. For example, predicting risk for mental health conditions could change patient care trajectories.

*There’s still quite a large stigma and gap in the need for mental health services ..we need to be very, very careful that when we’re developing these models…that providers…are using it in a way and like talking to the patient in a culturally appropriate manner and making sure that not a bad thing*. - DV03

Patients specifically expressed fear that risk predictions of mental health issues could lead to child protective services (CPS) interventions.

*I may run a 100 score and it’s like, ‘oh i passed’. Like, why is the doctor coming to talk to me? And ACS* (local CPS department) *is coming to take my child?* - PT12

Health professionals also raised the concern about model bias, noting the importance of utilizing representative training data and valuing fairness equally to accuracy.

*I’m sure they’d be curious if their ethnicity is put in there…But what if there is some type of bias in the tool?* - HP02

Patients and health professionals viewed AI as potentially harmful due to the stress that high-risk predictions may cause patients. Developer participants described the importance of involving health professionals and patients in the early stages of AI development to identify and mitigate these harms.

*The patient is going to develop that insight and be on board… and lead to like a more … patient-centered treatment*. - DV08

This convergence across groups on harm mitigation suggests that harm reduction may function as a shared value that could anchor implementations. However, the specific harms prioritized differed: patients focused on stigma and unintended consequences in other, non-health-related areas of life, health professionals on bias, and developers on upstream mitigation during development.

### Clinical benefit and utility

All groups expressed that AI must provide clinical benefits to patients, care partners, health professionals, or health systems. While developers may not necessarily be involved in reaping these benefits, they agreed that it was the ultimate purpose of their work. All groups agreed that for models to provide utility, a health professional or patient must be able to easily take action (e.g., making a referral, prescribing a medication, contacting their provider) based on the AI’s output.

*In terms of who can use the model, who benefits from the model, who can use the model and the insights. It’s completely focused on families, patients and health care systems… The insights for the individual are the primary focus and what they can do with that information. And so the idea is that that information will be most useful if it is talked about and and is actionable*. - DV09

Translating predictive models into action also requires a threshold for action. Patient participants, however, had challenges understanding what the numeric risk predictions meant practically and why there were certain thresholds for action (e.g., the tool recommended a mental health referral if the PPD risk was >30%).

*0 to 30%, you know, that’s a closer gap. And then you have like this gap from 32 to 100, but it’s like 40, 50, 60, 70. And I’m assuming the prevention treatment is starting at either 75 or 80. And I was like, what about those people in the middle?* - PT12

Health professionals highlighted the importance of clear, explicit recommendations. This uncertainty reflects that even when risk predictions are trusted, the absence of clear action pathways limits their clinical utility.

*If I’m a nurse and I see this, it’s kind of like, okay, that 32%, they’re not super high, but they are above the recommended threshold for treatment. But what do I do with this information? Do I call the O.B.? Do I call the social worker? Do I call psych?* - HP08

To trust AI and promote relevant actions, most patients and health professionals wanted to understand the accuracy of AI. Ideally, patients wanted to know how accurate it was for them individually. However, some patients did not need to see this information themselves but trusted health professionals to make judgments on when AI was safe for clinical use.

*I feel like if you’re showing me this and you’re saying like Oh it’s about 75% accurate, I may take heed to it a little less than if you said like a 90%*. - HP08

*I assume that [if] it’s in my patient portal, I’m thinking that it’s accurate for me*. - PT01

Relatedly, developers noted a tension between high performance and making understandable models that people wanted to use. This tension between accuracy and explainability illustrates a fundamental divergence: developers framed it as a technical tradeoff, whereas patients and health professionals experienced it as a barrier to trust and action.

Interviewer: *Accuracy versus understandability and explainability, and if you could only choose one, which one would you choose and why?*

DV01: *We need both, you can’t really do it without both, but when forced…the majority of the crowd choose understandability*.

Developers viewed implementation and ensuring utility as a continuous process that would require updates over time, similar to any traditional software. Developers also acknowledged the importance of end-user feedback throughout AI model development. Although they did not always feel they had the skills to include this feedback, particularly related to patient users.

*I think one piece that’s definitely missing in my research is patient perspectives about whether they think this kind of tool would be useful…I think that those questions have to be asked very carefully…But I don’t know that developers should be the ones actually going in to ask those questions*. - DV07

### Communicating AI models

Stakeholder groups agreed it was important to consider if, when, where, and how AI-related information would be communicated to patients. Patients had differing perspectives regarding when they would like to view AI output, with some preferring to see it before clinical encounters and others wanting someone to explain it to them. They requested various options for personal access, including through the patient portal, on mobile phones, via PDF, and secure email. Some also wanted the ability to share the information with chosen care partners, but this varied from person to person.

*I’ll assume these evaluations are not something that’s just sent. I feel you need to console them so I feel they should come to the hospital, be seen in the office, sit down, talk to them, explain the chart carefully and tell them everything, the risk factors and everything. -* PT04

*I personally think that the portal first cause then, like, that sort of like prepares you…a lot of times, you go to the doctor’s office and, you don’t really have time to contemplate things. -* PT10

All groups also highlighted that patients would need support in interpreting information and suggested that the provider may be the best person to do this. However, they also noted that the provider may need training and dedicated time to do this effectively.

*It’s probably not healthy to just tell someone they are sad or like tell someone that they have like a specific symptomology or anything like that…I think there’s a lot of work to do around that and like fostering trust in that setting…I think that’s where trust needs to lie with the clinicians*. - DV04

*Who gets the alert, and what happens once you get an alert? If you’re going through all that, then how much time are you spending to explain it?* - HP06

Developer and health professionals highlighted that interfaces should be presented in lay terms, accessible to different audiences. Feature importance was discussed at length - health professionals suggested conveying predictors of risk (e.g., medications, history of past illness) as both positives and negatives contributing to the patient’s total risk.

*Stakeholders…can handle…different amounts and types of information about what’s going into a model…But they have different needs in all different ways in terms of the quantity, the type, the way it’s communicated, everything about it could be different*. - DV06

*We tend to kind of speak in negatives or exception, where we only identify what’s wrong and not what’s right. I think it’s really important to identify the positives, too, if you’re going to show this to the patient*. - HP05

Patients generally wanted to see all features, not only the modifiable predictors. Providing sensitive features (e.g., risks related to marital status, BMI) requires care but may stimulate educational conversations. It was also important to ensure the labels were understandable by patients, so using the EHR-based feature names (e.g., “thyroid preparations”, “hyperemesis”) may be insufficient.

*No, I think they’re all helpful. I would maybe change the wording*. - PT12

### Data security, privacy, and access

All stakeholder groups agreed that the way the data is stored, the algorithm is computed, and displayed to end users must prioritize patient privacy and safety. AI developers, however, highlighted a tension between making tools open source and protecting patient privacy, ensuring their autonomy over their data. This tension between data utility and privacy protection was a recurring concern among developers, who felt responsible for navigating competing demands for model transparency and patient confidentiality.

*I think it’s really hard…to have data that is both not identifiable and still useful*. - DV04

Across groups, accountability was desired but diffusely assigned; patients looked to providers and institutions, health professionals sought clear role delineation, and developers emphasized shared responsibility. Many viewed external organizations, such as the Centers for Medicare and Medicaid, as having a responsibility for ensuring privacy (i.e., data de-identification) and also governing privacy rules, although it was not always clear as to which entity was responsible. Health organizations were also mentioned as having a role in supporting data privacy and promoting data access. Again, there were other external organizations whose access to data (e.g., CPS/ACS) was viewed by all stakeholders as detrimental.

Interviewers: *do you think there’s anything that they would need to know about privacy for the patient?*

Interviewee: *I mean, it’s a given, right. HIPAA. Every time you have a provider and patient conversation that is bind by HIPAA*. - HP06

*Because the ACS is not really functional…but the way that it’s been functioning, it causes a lot more like a threat to the patients access to that service rather than as being helpful*. - HP04

All stakeholder groups further highlighted how the combination of perinatal and psychiatric concerns made privacy issues more sensitive.

*Whenever a pregnant woman talks about this type of information…that concerns like calling to Child Protective Services. That is a big privacy issue…clinicians worry about models raising severe alerts*. - DV09

Patients expressed nuanced preferences for data control that varied by individual circumstances. Some wanted autonomy over when and when to share predictions with chosen care partners, while others preferred providers to determine appropriate information sharing. Notably, patients had limited awareness of or opinions about formal governance structures, focusing instead on practical concerns about who could access their data in real-time clinical scenarios.

*Personally, I think it’s better if I see it just by myself*. -PT11

### Responsible AI practices and liability

Stakeholder groups had various opinions about who was responsible for the safe development and implementation of AI. Participants mentioned responsible entities at various levels, from insurers and device manufacturers to individual developers and health professionals.

*There needs to be some responsible party who’s well informed, who’s making the decisions about if and how these tools are actually used and up to date on how well they’re actually being used in patient care as you introduce them* - DV08

Many of the questions of responsibility stemmed from a desire to mitigate individual liability for health professionals. The prevailing perspective from developers and health professionals was that no single person or entity could be held responsible, and a structure of shared responsibility was necessary. Patients perceived that determining safety for the use of an algorithm should fall to the health system or their health professional. However, most patients focused on immediate accountability, specifically, who would help them if a prediction was wrong or caused distress. When asked directly about participating in AI oversight, patients expressed interest but uncertainty. Health professionals also highlighted the need for responsibility in terms of seeing and acting upon risk predictions. Developers, on the other hand, felt responsible for making systems with high predictive performance that also mitigated potential biases.

*I assume that [if] it’s in my patient portal, I’m thinking that it’s accurate for me*. - PT01

*So a clinician needs to make an informed decision. In which case…the clinic is liable, as they would be for any misdiagnosis or incorrect treatment decision*. - DV08

*If we implement it then…somebody has to have ownership to it that you are responsible for this. And if the patient is seeing this information, somebody has to interpret for them*. - HP06

Responsible practices mentioned included checking for bias and accuracy prior to implementation, as well as continuous monitoring to identify model drift and retrain models. The conversation surrounding ensuring accuracy could be challenging, however, as the gold standard for comparison may not always be clear.

### Data quality

Health professional participants described challenges related to ensuring data quality, primarily related to bias, missingness, out-of-date information, and shortcomings in interpreting unstructured data (e.g., clinical notes) with insufficient context.

*These days…charts are not well updated… unless it’s thoughtfully updated, which it rarely is because hospital systems are far too busy for that kind of meticulousness, then I think that people are going to feel like I could see people being like, ‘where the hell did that come from?’* - HP03

Developers and health professionals both noted that missingness was particularly problematic in a perinatal context, where many may receive care for other specialties in different health systems.

## Discussion

Our findings suggest that AI implementation in health systems may require coordination between patients, health professionals, developers, and health systems administrators and leaders. Through interviews with nearly 40 stakeholders representing these perspectives, we identified requirements for the implementation and use of patient-centered AI systems.

We summarize our findings in preliminary requirements derived directly from stakeholders, shown in Table 3. The requirements identified vary considerably in both severity and solution complexity. Health professional competencies and stakeholder engagement in development represent severe but tractable challenges. Solutions exist through established educational pathways and participatory design methods. However, they require resource allocation and cultural shifts in AI development practices. Effective communication of AI output and patient-centered communication strategies present moderate complexity, requiring advances in explainable AI, user interface design research with diverse populations, and flexible technological infrastructure to accommodate varying patient preferences and health literacy levels. AI governance structures represent the most severe and complex challenge, with unclear accountability, regulatory gaps, and liability concerns requiring coordinated effort across federal agencies, professional societies, and individual institutions.

**Table 3:** Stakeholder-driven requirements for patient-centered AI

Topic	Requirement description	Related themes
Health professional competencies related to AI	Provide health professionals with the time and training to deliver AI-based risk prediction to patients	Mitigating harm, communicating AI, and responsible AI practices
Ethical creation of AI systems	Engage health professionals and patient stakeholders *throughout* the development process	Mitigating harm, clinical benefit, and utility
Effective communication of AI output for all stakeholders	Promote actionability by displaying options for viewing model accuracy, predictors, and thresholds for action	Mitigating harm, communicating AI, and responsible AI practices
Patient-centered AI communication	Include flexible options for communicating AI output to patients to manage cultural preferences and varying literacy levels	Mitigating harm, communicating AI, and responsible AI practices
AI governance	Making ethical decisions related to governance issues, including privacy, access, liability, and regulation, will require multiple levels of stakeholder engagement	Data security, privacy, and access; responsible AI practices and liability; AI regulation

Based on our use case, these findings pertain directly to predictive algorithms, and broader applications to generative AI systems or agentic AI tools should be explored. Our results, specific to end-user perspectives on conveying AI-based risk predictions of PPD overlap with Williams et al.’s recent qualitative study on a similar topic^22^. Several of our findings are likely condition-specific, such as concerns about CPS involvement, the intersection of perinatal and psychiatric stigma, and the time-bounded nature of the pregnancy window. However, other findings, including the need for actionable outputs, flexible communication strategies, shared governance, and health professional competencies, reflect implementation considerations that are broadly applicable across clinical AI contexts and patient populations. However, our work extends upon their findings, incorporating developer perspectives and using PPD as a use case to make broader recommendations for patient-centered AI regardless of clinical context. Our findings also resonate with the recently released AI Code of Conduct for Health and Medicine from the National Academy of Medicine, related to: advancing benefit for humanity, ensuring equity, engaging impacted individuals, ensuring workforce wellbeing, monitoring performance, and supporting continuous learning^23^. We describe practical next steps with data-driven insights for achieving these requirements across key stakeholder groups.

First, health professionals will require competencies to use AI effectively and safely in healthcare^24^. Most patients reported that they wanted their health professionals to deliver predictive algorithm results, whereas most health professionals do not feel qualified to do so. Health professionals also highlighted the difficulty in interpreting AI output and the limited time during encounters. While some efforts have attempted to integrate AI competencies into medical and nursing education^24^, far more work is needed to educate the existing workforce of health professionals. It may be efficient and beneficial to explore how pairing large language models (LLMs) with predictive algorithms may support this process. For example, many health systems are assessing LLMs for drafting secure portal message responses^25,26^. Future work may investigate how LLMs may be integrated into patient-facing tools (i.e., the patient portal) to provide patient-centered explanations of clinical concepts. Additionally, future work should also seek to improve AI literacy for patients interacting with AI outputs, as well as clinical context literacy for developers to ensure models reflect real-world care delivery considerations.

Second, our findings highlight how decisions made during the development process can have downstream impacts, for example, balancing explainability with accuracy, or addressing fairness. Model developers, at times, work in relative isolation from the health system leadership, health professionals, and patients who may ultimately adopt their model. Technical development is viewed as a foundational step with little input needed from other stakeholders. Rather, our findings suggest that because of the downstream implications of decisions made at the modeling stage, health professionals and patient stakeholders should be included in the early stages of development and evaluation to ensure models prioritize the right values, are useful, and ethically sound.

Participants also raised issues related to how data completeness and timeliness may affect model accuracy. Similar to displaying significant model features, visualization may support end users in understanding when key data elements may be missing or outdated. A systematic review of 44 implemented predictive AI models highlighted the lack of attention to user workflow and providing risk predictions in a way that enhances rather than degrades cognitive processing^12^. Future work may investigate generalizable best practices for displaying predictive AI models to work collaboratively with human end users.

Third, to provide clinical utility, predictive AI output needs to be actionable, as we have previously reported^8^. For example, perinatal providers need a way to refer appropriate patients for psychiatric or social work evaluation. Participants noted several needs to promote actionability, including providing reasoning (i.e., risk factors) and the level of model accuracy. This information would allow them to weigh their expertise in conjunction with the model and choose from multiple possible actions based on the patient’s risk factors. Patients wanted to understand individual risk factors, which could support shared decision-making conversations with providers regarding risk reduction. Participants also noted that meaningful thresholds for action based on the numeric risk prediction were needed. This is not done consistently in practice, and there is a lack of robust methods for determining these thresholds for actionability. As illustrated by participant responses in our study, even small differences near a threshold boundary (e.g., 32% vs. the 30% cutoff) created confusion about appropriate clinical action, underscoring the need for clear communication about threshold rationale and the management of near-threshold cases. Including confidence intervals or uncertainty indicators alongside risk predictions may further support clinical decision-making by helping end users calibrate their trust in individual predictions, as stakeholders in our study emphasized the importance of understanding prediction reliability.

Fourth, patients in our study expressed a wide range of preferences and information needs related to the interpretation of the model. In fact, several of our core recommendations (i.e., flexible communication options, lay-language interfaces, and the inclusion of modifiable risk factors to support shared decision-making) were derived directly from patient-identified needs and preferences. Compared to health professional end users, less attention has been given to patients as end users of models. There are many scenarios in which patients may view model output, making their perspectives vital in considering how to display output. The harms of failing to incorporate their perspectives may include elevated worry and anxiety, increased outreach to care teams, and even unnecessary care visits.

Lastly, several other publications have provided detailed needs assessments for enhancing privacy, navigating liabilities, and devising regulations related to AI^23,27–30^. Our findings reinforce that layers of shared governance, involving multiple stakeholder groups, will be necessary to address these challenging questions.

Our findings both replicate and meaningfully extend previous systematic reviews. Consistent with prior work, we found that stakeholders acknowledge AI’s efficiency and data processing advantages while expressing concerns about transparency, bias, and the potential for AI to affect human communication^11^. However, our integrated approach reveals how these concerns manifest differently across stakeholder groups, with patients emphasizing personal autonomy and the preservation of human elements in care, health professionals focusing on workflow integration and liability, and developers prioritizing technical performance while recognizing the need for end-user engagement. Others have identified the importance of contextualizing risk predictions and understanding effective information sources, yet reported that minimal evidence on actual communication strategies or clinical outcomes exists beyond 6–12 months^7^. Our work addresses this gap by providing concrete, stakeholder-informed recommendations for how risk predictions should be formatted, delivered, and acted upon. Similarly, while others have documented that patients find AI decision aids user-friendly and empowering, they also noted concerns about accuracy, data privacy, and the risk of over- or under-treatment^19^. These themes that emerged strongly in our interviews and that we extend by proposing specific governance structures and communication protocols to address these concerns. A critical contribution of our work is demonstrating that effective AI implementation requires not just addressing each stakeholder group’s concerns independently, but creating systems that align their sometimes divergent needs and expectations.

Our findings also suggest multiple important areas of future work. First, our study primarily focused on predictive AI models. Generative AI is being rapidly adopted in research and in healthcare settings. A recent systematic review identified over 200 studies that have used LLMs for healthcare purposes, including patient education, recommendations, and guidance, customizing care plans, interpreting medical information, and facilitating communication between patients and health professionals^31^. They reported positive findings relating to patient satisfaction but concerns about readability, accuracy, and bias. Thus, investigating patients’ needs and ability to use LLMs safely will be an important part of ensuring appropriate use in healthcare.

Additionally, the science for both predictive algorithms and generative AI has weighed heavily towards model development and validation, with significantly fewer studies evaluating real-world clinical implementation and health outcomes. Multiple perspective pieces have called for more implementation science studies^32^, along with clinical trials that have thoughtful comparator arms and benchmarks representing the current state of the healthcare system^33^. While improving clinical outcomes is the ultimate goal for most predictive AI systems, recent studies have highlighted that only measuring these outcomes may produce an incomplete picture, particularly when AI does not have the intended effect^34^. We advocate for detailed tracking (e.g., via EHR log data) that may ascertain how AI impacts decisions, how it is accessed by patients, and the downstream impacts this may have on patient care. All of these metrics may not be assessed in a detailed manner, but having them available supports investigating “last mile” problems when highly accurate AI fails to improve clinical outcomes. This evidence is critically needed to inform continued surveillance and refinement of AI models and to guide strategies for operationalizing the broad guidelines and frameworks that professional bodies such as the National Academy of Medicine have published^23^.

Finally, there are notable gaps in patients’ expressed interest in understanding more details about model predictions, and the strategies that we presently have to provide this information in a clear and comprehensible way. Despite substantial interest in the explainability of AI, very little explainability work has considered patients as end users^35^. Health professionals themselves may also struggle to determine how and when to follow AI recommendations, let alone how to deliver this information to patients^36,37^. These findings point to the need to both equip health professionals, including nurses and providers, with AI-related competencies or “literacy”^36,37^.

Limitations of this study include that we based part of our interview guide on an existing algorithm predicting PPD; thus, findings may not generalize to other clinical contexts or populations. Data access policies vary by institutional and regulatory context, which may influence both patient and provider engagement with AI-generated outputs. Additionally, though we strived to invite a diverse range of stakeholders to participate in interviews, some perspectives may have been underrepresented. Specifically, we oversampled patient participants, which may have led our recommendations to be more considerate of patient needs than those of health professionals and developers. This imbalance may have also influenced theme prominence, with patient-salient topics (e.g., communication preferences, anxiety about predictions) potentially receiving greater emphasis relative to developer or health professional concerns such as workflow integration and technical performance. Although our purposive sampling approach was necessary to obtain broad representation across roles for health professionals and clinical domains of expertise for AI developers, our individual outreach through personal contacts may have introduced unintended biases. Additionally, the limited racial and ethnic diversity within each stakeholder group constrains our ability to detect differential perspectives by race or ethnicity. Future work should prioritize recruitment strategies that enable examination of how these factors shape preferences for patient-centered AI. Thus, future work building upon these findings to further investigate patient-centered AI across a range of clinical contexts and populations, with additional balanced stakeholder groups, is needed.

## Methods

### Setting, study design, and sample

We conducted prospective qualitative interviews with AI developers, health professionals, and perinatal patients. Health professionals and patients were potential end users of the tool in our use case, and AI developers had relevant expertise but were not directly involved in the development of the given tool. Table 4 describes the inclusion criteria and recruitment methods with additional details in the Supplementary File 2.

**Table 4:** Inclusion criteria and recruitment sources by participant stakeholder group

Stakeholder group	Inclusion criteria	Recruitment sources
AI developers	- Involved in the AI technical development process - Had experience with AI applications in mental or reproductive health - English-speaking	Professional organizations (e.g., the American Medical Informatics Association) and personal connections
Health professionals	- Provides care to perinatal persons (related to somatic care, mental healthcare, or both) in the U.S. - English-speaking	Departmental listservs, personal connections, snowball sampling
Patients	- Currently pregnant or within 1 year postpartum - Received/receiving perinatal care in the U.S. - English-speaking	Social media (Instagram, Reddit, Facebook), Columbia University (RecruitMe, clinic fliers)

All participants provided written informed consent. The Columbia University Institutional Review Board approved the study (AAAU3398).

### Underlying algorithm and use case

We based our use case on a machine learning model created by members of our research team for proactively predicting PPD during pregnancy. The model is intended for use during pregnancy to identify patients at elevated risk of PPD before symptom onset, enabling proactive referral to psychiatric or psychosocial support services. We created the initial model using EHR data from a single site comprised of 32 clinical features, including elements of mental health history, medical comorbidity, obstetric complications, medication prescription orders, and patient demographic characteristics. The best performing model used data through childbirth and used logistic regression with L2 regularization, achieving an area under the curve of 0.937^38^. In preparation for clinical implementation, we updated the model with additional years of EHR data and evaluated it against data from a clinical research network database, including a more diverse patient mix from multiple institutions. This allowed us to revise the model to balance performance, fairness, and clinical appropriateness of the included features. This analysis revealed that a risk threshold of provided the best balance between true and false positives^39^. We provided information to participants as specified and used this model as a reference for questions from participants regarding the nature of the model and the data used to create it.

### Data collection

Our team iteratively developed interview guides (Supplementary File 1), combining insights from MITRE’s Ethical Framework for the Use of Consumer-Generated Data in Health Care and Sittig and Singh’s Socio-technical model^20,40^. Experts in obstetrics, perinatal mental health, nursing, AI development, consumer informatics, and qualitative methods provided input. We piloted each guide before enrollment. We showed patient and health professional participants a mockup of how the algorithm from our use case (i.e., proactive prediction of PPD) may be displayed to concretize the discussion. Table 5 describes preliminary information from the interview guide provided to patient participants immediately prior to displaying the mockup.

**Table 5:** Definitions and descriptions provided to participants regarding background information and the predictive algorithm

Type of information	Description provided to the participant
Definition of medical records and electronic health records^43^	*You’ve probably seen your chart at your doctor’s office. In fact, you may have charts at several doctors’ offices. If you’ve been in the hospital, you have a chart there, too. These charts are your medical records. They may be on paper or electronic. To keep track of all this information, it’s a good idea to keep your own personal health record.An EHR (electronic health record) is a computerized collection of a patient’s health records. EHRs include information like your age, gender, ethnicity, health history, medicines, allergies, immunization status, lab test results, hospital discharge instructions, and billing information*.
Description of artificial intelligence^44^	*People have a lot of different ways to describe artificial intelligence or “AI”. One way to think about it is as a computer system that can do or mimic tasks people usually do, such as reasoning, learning from examples, communicating, displaying or understanding emotions, and planning and making decisions*.
Outside of AI in clinical care	*As we mentioned, one kind of AI involves learning from examples. In healthcare, there are some teams that use computers to look at large amounts of past electronic health record data to find patterns in patients who had a certain disease or experience. For example, they may look at electronic health record data of women who were eventually diagnosed with breast cancer to find patterns or common risk factors. Then, the patterns that are learned can be used to look at someone’s current information and guess or estimate their risk of a disease, such as breast cancer*.
Introduction to specific use case	*We would like to ask a few more detailed questions about this so I am going to show you an example of what a tool that uses AI to estimate the risk of postpartum depression*. *This is just an example - this is not a real patient, but the tool would allow us to guess the risk for someone experiencing postpartum depression during their pregnancy. We are going to imagine we are looking at a risk score for a patient named Cynthia who is 35 weeks pregnant. She visits her doctor for a checkup, who shows her a screen with this on it*.

**Fig. 1:**
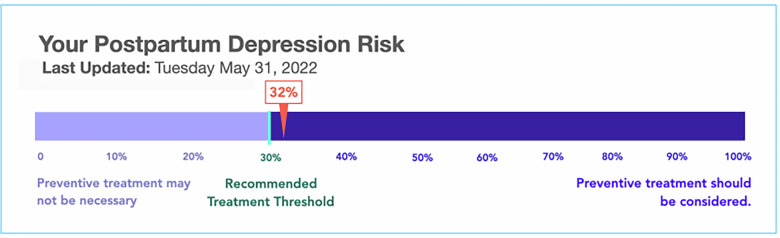
Sample output of AI-based postpartum risk prediction tool shown to patient and health professional participants. We provide a “last updated” section to signify that the prediction may change over time. The risk prediction is presented on a number line based on a previous study of participant trust and preferences in different visualization formats^41^. We utilize 30% or 0.3 as a cutoff for treatment recommendation based on our empirical work developing the predictive algorithm^39^. As the algorithm predicts PPD, but is intended for use during pregnancy, the action labels focus on preventative treatment and were iteratively developed with our team’s expertise in the AI algorithm, obstetrics, and perinatal psychiatry.

The mockup was informed by a survey of over 500 perinatal participants^41^. We found that participants trusted AI-based risk scores that included a concrete risk number (instead of phrases like “above average”), and that participants preferred number line graphics over numbers alone (Fig. 1).

Three researchers trained in qualitative methods conducted all interviews via Zoom or telephone (NCB, PMD, ZR). Interviews lasted 30–60 min and were audio recorded. Participants were compensated $50. Following the interview, participants electronically completed a demographics survey.

### Data analysis

We conducted a directed content analysis using thematic analysis and the constant comparative process, employing a combination of deductive and inductive coding^42^. In this paper, we focus on insights derived from the dimensions of the Sittig and Singh Socio-technical Model. which we first used to deductively organize the data, but with adapted definitions and the addition of sub-codes to suit our application to predictive AI^20^. A group of three coders (NB, PD, ZR) first analyzed transcripts collaboratively. Two team members then coded each transcript independently into the pre-specified dimensions of the Sittig and Singh Model. We then met to compare and resolve disagreements, using the third coder in cases where agreement could not be reached. During the coding process, we also evaluated thematic saturation across stakeholder groups. We defined saturation as the diminishing need to: (1) update existing parent code definitions to match our use case, and (2) add or revise sub-codes. We monitored saturation iteratively across the full sample and within each stakeholder group. In the final three interviews per group, no new parent codes emerged, and sub-code revisions were minimal, suggesting adequate thematic coverage. Once all transcripts had been coded based on the Sittig and Singh, we reviewed the data dimension-by-code to mitigate code drift. One of the original coders (NB) and another team member (MRT) then reviewed each parent dimension, inductively allowing themes to emerge that cut across the domains of the Sittig and Singh framework.

## Supplementary information

Supplementary Information

## Data Availability

All raw data are available for sharing upon reasonable request to the corresponding author.
