# Supplementary material for: A qualitative interview study investigating patient, health professional, and developer perspectives on real-world implementation of patient-centered AI systems
Source: NPJ Digit Med. 2026 May 5;9:352. doi: 10.1038/s41746-026-02587-5 (PMC13144722; doi:10.1038/s41746-026-02587-5)
Supplement: Supplementary file 1 — Supplementary Information [file 41746_2026_2587_MOESM1_ESM.pdf]

Supplement 1.1 - Patient interview guide

Background:

Do you use a patient portal?

As far as you know, does your healthcare team (doctors, nurses, etc.) maintain your medical information in an electronic format?

[Depending on the answer, can provide some of the following information from Medline Plus]
"You've probably seen your chart at your doctor's office. In fact, you may have charts at several doctors' offices. If you've been in the hospital, you have a chart there, too. These charts are your medical records. They may be on paper or electronic. To keep track of all this information, it's a good idea to keep your own personal health record."

"An EHR (electronic health record) is a computerized collection of a patient's health records. EHRs include information like your age, gender, ethnicity, health history, medicines, allergies, immunization status, lab test results, hospital discharge instructions, and billing information."

Have you heard of, or do you know about, different ways the information in your electronic health record is used?

Have you heard about "artificial intelligence" being used in healthcare?

- What does this term mean to you?

People have a lot of different ways to describe artificial intelligence or "AI". One way to think about it is as a computer system that can do or mimic tasks people usually do, such as reasoning, learning from examples, communicating, displaying or understanding emotions, and planning and making decisions

- How does this description make you feel?

Specific use case:

As we mentioned, one kind of AI involves learning from examples. In healthcare, there are some teams that use computers to look at large amounts of past electronic health record data to find patterns in patients who had a certain disease or experience. For example, they may look at electronic health record data of women who were eventually diagnosed with breast cancer to find patterns or common risk factors. Then, the patterns that are learned can be used to look at someone's current information and guess or estimate their risk of a disease, such as breast cancer.

We would like to ask a few more detailed questions about this so I am going to show you an example of what a tool that uses AI to estimate the risk of postpartum depression.

This is just an example - this is not a real patient, but the tool would allow us to guess the risk for someone experiencing postpartum depression during their pregnancy. We are going to imagine we are looking at a risk score for a patient named Cynthia who is 35 weeks pregnant. She visits her doctor for a checkup, who shows her a screen with this on it.

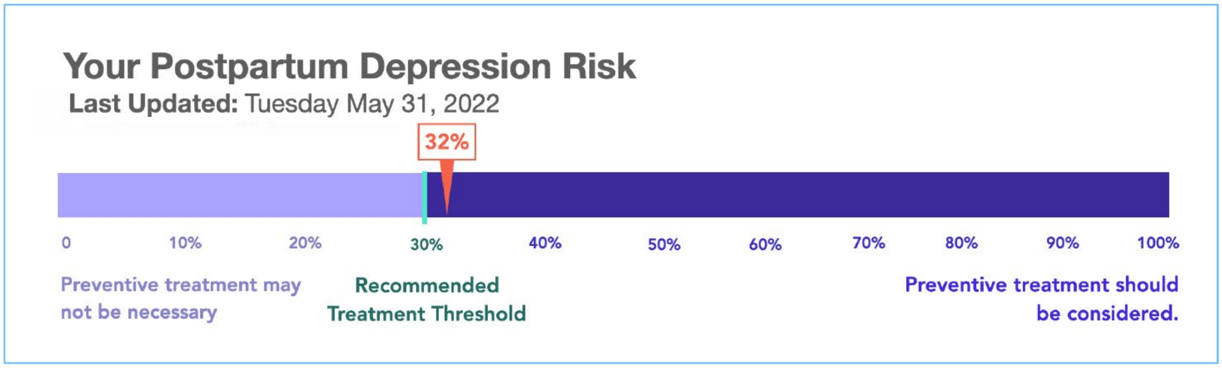

- What general feedback do you have about this screen?
  - What is confusing for you? What do you wish was clearer?
  - With this in mind, what are your general attitudes towards your doctors and others taking care of you in this way?
    - Positive, negative, neutral?
    - What comes to mind?
    - Would you want them to have asked permission first to calculate this risk score (give your permission)?
    - What would you want to know about privacy and how the data is used?
    - What would you want to know about security (how the data is stored and kept safe)? [Privacy and data protection]
    - What concerns would you have?
      - About the relationship with your doctor?
      - About the tool giving an incorrect recommendation/wrong answer?
- What is the right amount of information you would want to see?
  - What do you think about how this information is shown?
  - What do you think someone should do if this is their risk score?
  - Would you change anything about how the information is presented?
  - Is the graphic helpful?
  - Do the words in the graphic make sense?
  - Is including the actual numbers (e.g., 32%) instead of just words (e.g., high, medium, low) helpful or not?
  - Would you want to see information about how accurate the risk score is?
    - How would you want to see this (e.g., percent accuracy, a graph, just a notification that someone had made sure it was accurate)?
  - Do you want to see this screen or only have a conversation with your doctor?
  - Do you want to be able to print or access this screen at home, and why or why not?
  - Do you want to see all risk factors the tool picked up on, or only the modifiable ones [the ones that can be helped or changed]?
  - What is more important to you - that the tool can explain how it got its suggestion or that the tool's suggestion is accurate?
  - Would you want to know more about if or how the tool worked for different groups of people?
  - Are there specific scenarios in which you would want more or less information?
- Imagine this is your risk score. If this was being shown to you, how open would you be to:
  - Self-monitoring your mental health more closely

        - Would you be open to self-tracking symptoms using a mobile app? Why or why not? How often would you be willing to track?
    - Using app-based therapy (asynchronous - not with other people)
    - Starting therapy - either in a group or individually
    - Seeing a psychiatrist and potentially starting a depression medication
- What if the risk score was higher (for example 80%)?
- How would you feel if a doctor partially based their decision-making on these machine learning algorithms kind of AI we talked about?
    - What are some reasons for your positive or negative attitude towards it?
    - Would it change the trust you have in your doctor?
    - What are some ways doctors could increase your trust in an AI tool machine learning tool?
- Would you want to share this information with anyone (trusted friends or family members)?
    - How would you want to share this information?
    - What information would you want to share? For example, just the score, this visualization, risk factors we discussed, everything you have access to?

Supplement 1.2 - Health professional interview guide

Background

- To provide some context, please describe your current role as a health professional?
    - What are some of your specific responsibilities?
- Can you tell us where you work/practice?
- One thing we are focusing on this work is the identification of postpartum depression. What role do you play in identifying risk of PPD?
    - Is it an important part of your role? Why or why not?
    - Is there a process for identifying patients at high risk of developing PPD?
    - What does not work well about the existing process?
    - What does work well?
    - Are there other roles important to this process?

- What do you think of when you hear “artificial intelligence”? What does the term mean to you?
    - Do you use artificial intelligence or machine learning in your clinical practice [that you are aware of]? If so, how?

Specific use case:

Now imagine that there is an AI tool that can use patients' past health information to give them a risk of developing PPD. We want to understand if such a tool were to exist, how it could be most useful. We will ask for your thoughts about this idea and attributes that may be important to consider in development. Next we are going to show you an example of how this could be presented to a patient. This is just an example - this is not a real patient, but the tool would allow us to guess the risk for someone experiencing postpartum depression during [or after] *their pregnancy. We are going to imagine we are looking at a risk score for a patient named Cynthia who is 35 weeks pregnant/is bringing their infant in for their one-month well-baby checks. Cynthia visits their baby’s doctor for a checkup, who shows them a screen with this on it.*

- What questions would you have about an AI algorithm that helped predict risk of postpartum depression?
    - How much do health professionals needs to understand about how an AI tool works?
- Is this something you could see yourself using with your patients' parents? Why or why not?
- Do you think the benefits of such an AI tool outweigh potential harms? Why or why not?
- How can such an AI tool help patients understand their care options, if at all?
    - When would it be appropriate for this information to be shared with the patient? [If at all] (e.g., before a visit, during a visit, after a visit)?

    - Where do you think it could be viewed? (e.g, patient portal, print out in the office, secure email)
    - Are there logistical, technical, legal, or ethical challenges with this information sharing? Especially since the child is your patient, not the parent?
    - Are there certain groups of patients this would be more or less appropriate for (e.g., lower risk vs. higher risk)?
- Beyond the result (their risk for PPD), what elements of the AI tool is it important for patients to understand?
    - Is it important they understand the factors that are driving this risk? What about factors that are non-modifiable (e.g., previous history of depression) or sensitive (e.g., being unpartnered)?
    - Is it important for them to know the accuracy of the model? Or just that is has been validated, reviewed by their provider?
    - Is it important that they know about any biases in the model? Or should biases simply be removed before use?
- How do you think this tool could impact patient trust in you or other members of the care team?
    - What kinds of options would you want to have if you disagreed with the tool's predictive risk?
- What concerns would you have about the privacy of patient data related to AI? What about your privacy as a provider?
- How much do you think patients need to know about if/how AI is used to make decisions in their care?

Is there anything else that is important to you from an ethical perspective?

<u>Supplement 1.3 - AI developer interview guide</u>

Background

- What is your professional experience with AI and how it could be used in healthcare?
    - What applications have you worked on related to reproductive health or mental health?
    - What are the primary objectives of applications you have worked on (e.g., diagnosis, screening, prediction, etc.)? Generative v. predictive?
    - Who are the primary users of the you have worked on (e.g., researchers, clinicians, patients, other health professionals)
    - What data is used to develop the models you have worked on? How/from whom is this data obtained (e.g., EHR, claims, patient-generated).

Bioethics questions

Next, we want to ask you about some ideas and values developers often have to keep in mind and learn more about how you think about each idea in your work, if at all.

- Who do you think should have the autonomy to use the models you have worked on to make health decisions?
    - How do you think about this in the context of your work?
    - What information do you think is important for users to understand? Is it different depending on who the user is (e.g., researcher, provider, patient)?
    - Are there additional considerations about this in the context of mental health, reproductive health, or their intersection (e.g., post-partum depression)?
    - Do you think it would be helpful or feasible to allow patients to opt into AI-informed care?

- How do you consider if the benefits of a model outweigh the potential harms?
    - What information do you think is important for users to understand? Is it different depending on who the user is (e.g., researcher, provider, patient)?
    - Do you compare the performance of the model to current expert-human performance?
    - How do you also think about this in terms of the harms of misdiagnosis (e.g., suicide in the military context)?
    - Who do you think is/should be held responsible if misdiagnosis or harm occurs when a model is being used to treat a patient/patient(s)?
    - Are there additional considerations about this in the context of mental health, reproductive health, or postpartum depression?

- How important is it that the end users understand the model and model features?
    - How do you think about this in the context of your work?
    - What information do you think is important for users to understand? Is it different depending on who the user is (e.g., researcher, provider, patient)?

    - How do you think we currently do in helping diverse users understand…(probe about researchers, providers, and patients) the models that are created?
    - Are there additional considerations about this in the context of mental health, reproductive health, or postpartum depression?

- How important is it that the model improves trust in healthcare decision-making?
    - With whom are you trying to foster trust in the models you develop (probe about researchers, operations/hospital administration, providers, patients)?
    - What do you think your role is in considering how the model may affect the patient-provider relationship?
    - What information do you think is important for users to understand? Is it different depending on who the user is (e.g., researcher, provider, patient)?
    - Are there additional considerations about this in the context of mental health, reproductive health, or post-partum depression?

- How important is it that models consider privacy of data?
    - How do you think about this in the context of your work?
    - Whose privacy are you concerned about in your work?
    - What information do you think is important for users to understand? Is it different depending on who the user is (e.g., researcher, provider, patient)?
    - Are there additional considerations about this in the context of mental health, reproductive health, or post-partum depression?

- What does model transparency mean to you?
    - How do you think about this in the context of your work?
    - How do you think we are doing in terms of conveying?
        - If/how a model was used to make decisions?
        - Model performance - accuracy gains
        - Model explainability
        - Model fairness
    - What information do you think is important for users to understand? Is it different depending on who the user is (e.g., researcher, provider, patient)?
    - Are there additional considerations about this in the context of mental health, reproductive health, or post-partum depression?

Detailed Sample and Recruitment Procedures

Patients

Based on the interview's focus on patient-centered AI, we first recruited and conducted data collection with patients. We sought to sample a racially diverse group of patients to understand various perspectives related to the use of AI, specifically for postpartum depression. We focused primarily on racial diversity as opposed to other sociodemographic characteristics, given our previous findings that patient perspectives on AI for mental health varied based on race.

We recruited patients through a combination of social media pages and interest groups related to pregnancy, postpartum, and consumer research studies. We also posted study materials on an internal site called "RecruitMe" where our health system's patients can express interest in study participation and in the waiting rooms of obstetric clinics. All posts directed patients to an institutional-review board (IRB)-approved study flier that contained a link and a QR code to a Qualtrics form prospective participants could fill out to express interest. Fliers deliberately kept the purpose of the study vague, broadly stating that we were "conducting interviews to understand how we can support physical and mental health during pregnancy and postpartum". Participants were then informed during the consent process that they could stop the interview at any time or selectively not answer questions they weren't comfortable with. The interest form asked participants for preferred contact information, race/ethnicity, and questions that allowed us to confirm their eligibility. Qualtrics also passively collects participant IP addresses.

Prior to outreach, we first verified that participants had used a legitimate IP address and that they were located in the United States (one of the inclusion criteria). We then purposively reached out to groups of participants again, using IRB-approved materials, balancing a first-come, first-served model while also promoting racial diversity among participants. Interested participants then signed up for an interview time with a member of our research team. We provided participants a Zoom link but also conducted their phone number as a backup in case they were unable to use Zoom. We also provided participants the consent form so they may review prior to the interview. Study team members conducting the interviews first asked open-ended questions to confirm participant eligibility before proceeding to consent and study procedures.

Health professionals

We purposively sampled health professionals to obtain broad representation across professional roles, including obstetricians, midwives, nurses, social workers, and perinatal mental health specialists. To recruit these professionals, we utilized departmental listservs, outreach to individual persons representing the aforementioned roles by study team members and those within our professional networks, and snowball sampling with participants, also including IRB-approved recruitment materials. All outreach occurred via email. The outreach materials specified we sought "healthcare provider(s) who take(s) care of pregnant or

postpartum women in the U.S." and that we were conducting interviews "to learn more about how we can better support mental and physical health during pregnancy and postpartum. Specifically, we are investigating new technology-based interventions that may support well-being and improved maternal health equity".

Interested participants were provided with study team contact emails and could voluntarily reach out to set up an interview time. Similar to patient participants, study team members conducting the interviews first asked open-ended questions to confirm participant eligibility before proceeding to consent and study procedures.

Interviewers tracked and reviewed the roles represented by completed participants on a weekly basis to direct ongoing outreach that covered the roles of interest.

AI Developers

We sought to recruit those with broad involvement in the technical development of AI-based tools. Given the specificity of postpartum depression and the small number of developers working on this specific condition, we focused on sampling participants who had experience with AI-based tools for mental or reproductive health, to consider some aspects of the sensitive nuances associated with related conditions. We purposively sampled relevant roles to ensure we had representation from those with experience in each domain. Our sampling involved posting on relevant working groups within the American Medical Informatics Association, individual outreach through contacts of study personnel, and snowball sampling within participants. Outreach occurred through discussion board posts and email using IRB-approved materials. We deliberately took a broad conceptualization of developers, specifying to them in outreach materials that "we are reaching out to you related to your technology, algorithm, or AI development experience".

Interested participants filled out an interest form providing their contact information and preference, development domain experience in mental or reproductive health, and confirmed eligibility. Similar to the other two stakeholder groups, study team members conducting the interviews first asked open-ended questions to confirm participant eligibility before proceeding to consent and study procedures.

Interviewers tracked and reviewed the balance of clinical domains represented by completed participants on a weekly basis to direct ongoing outreach that covered the domains of interest.
